# Simulation and experimental investigation of liquid-cooling thermal management for high-bandwidth co-packaged optics

**DOI:** 10.1007/s12200-025-00156-4

**Published:** 2025-05-14

**Authors:** Senhan Wu, Song Wen, Huimin He, Jianyu Feng, Chuan Chen, Haiyun Xue

**Affiliations:** 1 State Key Laboratory of Fabrication Technologies for Integrated Circuits, Institute of Microelectronics, Chinese Academy of Sciences, Beijing, 100029 China; 2 Institute of Microelectronics of the Chinese Academy of Sciences, Beijing, 100029 China; 3 University of Chinese Academy of Sciences, Beijing, 100049 China

**Keywords:** Co-packaged optics, Optoelectronic integration, Thermal management, Liquid cooling, Manifold microchannel heat sink

## Abstract

This study explores the application of cold plate liquid cooling technology in co-packaged optics (CPO). By integrating optical modules and the switch chip on the same substrate, CPO shortens the electrical interconnection distance, effectively solving the problems of high power consumption and poor signal integrity of traditional pluggable optical modules under high bandwidth. However, the surge in power density and the thermal crosstalk resulting from high integration density make thermal management one of the key challenges that constrain the reliability of high-capacity co-packaged optics. For the unique architecture of CPO, this study analyzes its heat dissipation needs in detail, and a thermal management scheme is designed. The thermal management scheme is simulated and optimized based on the Navier−Stokes equation. The simulation results show that, in a 51.2 Tbit/s CPO system, the junction temperature of the switch chip is 97.3 °C, the maximum junction temperature of the optical modules is 31.3 °C, and the temperature difference between the optical modules is 2.4 °C to 1.2 °C. To verify the simulation results, a thermal test experimental platform is built, and the experimental results show that the temperature simulation difference is within 4% and the pressure change trend is consistent with the simulation. Combining the experimental data and simulation results, the designed heat sink can satisfy the heat dissipation demands of the 51.2 Tbit/s bandwidth CPO system. This conclusion demonstrates the potential of liquid-cooling technology in CPO, providing support for research on liquid-cooling technology in the CPO. The design provides a theoretical and practical basis for the high performance and reliability of optoelectronic integration technology in wavelength division multiplexing (WDM) systems and micro-ring device applications, contributing to the application of next-generation optical communication networks.

**Graphical Abstract:**

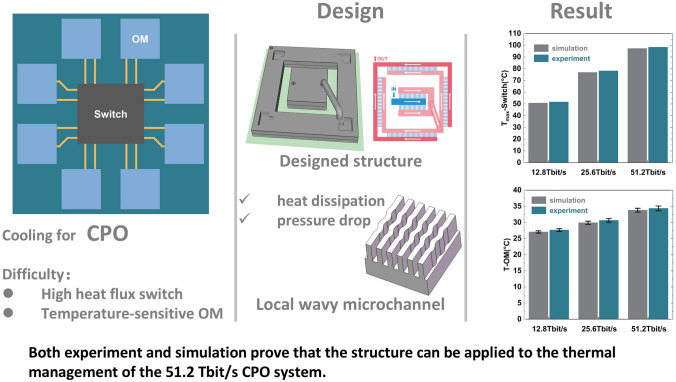

## Introduction

Currently, the artificial intelligence (AI) and machine learning technologies are developing rapidly, and data centers are facing significant challenges due to the surge in end-user numbers and the massive data exchange demand resulting from AI. To cope with this change, data centers need to improve data exchange speed and switch bandwidth to meet the increasing requirement [1–5].

In the data center, conventional optoelectronic interconnect technology usually uses board-side optical modules. However, as the bandwidth increases, the long electrical interconnect distance in the board-side optical module scheme leads to a series of issues such as signal quality degradation and increased system power consumption. According to Cisco’s data statistics, from 2010 to 2022, the power consumption of the switch chip increased by about 8 times, and in a 51.2 Tbit/s switch system, the total power consumption of optical modules will exceed 50% of the system power consumption [6]. Obviously, the traditional pluggable optical modules can no longer meet the requirements of data centers [7, 8].

In this context, there is an urgent need for a new technology, CPO (co-packaged optics). CPO integrates optical modules, which is also known as the optical engine, and the switch chip on the same substrate through advanced packaging technology, which shortens the electrical interconnection distance and effectively reduces the power consumption. In recent years, with the growing market demand, CPO has gradually shifted from the academic research stage to practical application.

In March 2020, Intel demonstrated the industry-first Ethernet switch product based on CPO, which combines a 12.8 Tbit/s Barefoot Tofino2 switch chip and 1.6 Tbit/s optical modules [9]. This breakthrough packaging solution marks a new stage in the application of CPO technology in data centers. And in 2022, Intel has once again introduced a 25.6 Tbit/s CPO prototype [10, 11]. The prototype realizes a high-density interconnection of five 5.12 Tbit/s TeraPHY Optical I/O modules from Ayar Laboratories to an FPGA chip by using EMIB (embedded multi-die interconnect bridge) technology [12–14].

In 2022, Marvel launched its 12.8 Tbit/s Teralynx 7 switch based on 2.5D CPO technology [15]. In the same year, Broadcom unveiled its first CPO switch at the Optical Fiber Communications Conference and Exhibition (OFC), which packaged the 25.6 Tbit/s Tomahawk 4 switch chip with optics [16]. In addition, Cisco is also investing in CPO technology to prepare for future transitions [17].

In March 2024, Broadcom released Bally, their 2nd generation CPO and also the first 51.2 Tbit/s CPO switch system, in March 2024. This system enables the optical interconnect to work with 70% less power than pluggable transceiver solutions, demonstrating the advantages of the low cost, high bandwidth, low transmission loss, and low power consumption of CPO technology [18].

In March 2025, NVIDIA released their first switch based on CPO technology, the Quantum-X800, which contains the CPO module integrating a 28.8 Tbit/s Quantum-X800 chip and six optical components. Each optical component consists of three optical engines to achieve 4.8 Tbit/s throughput. The pioneering application of a 200 Gbit/s micro-ring modulator in the optical engines reduces power consumption by a factor of 3.5. NVIDIA’s attempt means that micro-ring modulators may be used in more high-capacity CPOs in the future to achieve lower power consumption and higher bandwidth.

CPO technology has the advantages of low cost, high bandwidth, low transmission loss, and low power consumption. However, as an emerging technology, it also brings many new challenges, including fabrication difficulty, complex multi-physical field simulation, and reliability issues. Furthermore, the surge in power density and the thermal crosstalk resulting from high integration density make thermal management one of the key challenges that constrain the reliability of high-capacity co-packaged optics.

First, as the power of the switch chip increases [19, 20], it produces an extremely high heat flux in a high-capability CPO system. Insufficient cooling of the chips may lead to high junction temperatures, which can significantly degrade their performance and reliability [21, 22]. Therefore, a more efficient cooling solution is needed to replace the traditional air-cooled cooling [23]. In addition, tight mounting spaces in systems that can lead to significant fan performance losses and noise issues of air cooling are also reasons to select liquid-cooled cooling [24].

In addition, CPO integrates optical modules in close proximity to the switch chip, which may lead to thermal crosstalk between the switch chip and the optical module. The co-packaged optical devices usually have high temperature sensitivity, especially the micro-ring resonators, which are very susceptible to temperature fluctuations due to their precise structure [25, 26]. Therefore, it is particularly important to design a reasonable heat dissipation scheme.

Initially, CPO typically used air cooling [27]. For example, Intel’s first CPO product adopts air-cooled heat sinks to cool the switch chip, while heat pipe radiators are used to dissipate heat in the optical module section.

However, as the bandwidth continued to increase, the growth of switch chip power consumption challenged the thermal dissipation capability of air-cooled heat sinks.

In 2021, Intel made the first attempt to apply a liquid-cooled heat sink in the CPO. The junction temperature targets were 105 °C and 100 °C for the switch chip and the electrical chip of the optical module, respectively. The analysis covered both 32 °C and 37 °C inlet water temperatures, and the results showed that the junction temperatures of all the chips stayed within the allowable junction temperatures [4]. In 2022, the products demonstrated by Ragile Networks at the OCP (Open Compute Project) also used the liquid cooling cold plate design.

With the application of 51.2 Tbit/s switch chips, the traditional air-cooling method may not be able to meet the increasing heat dissipation demand of the CPO. Therefore, this paper explores the application of the liquid-cooled cold plate thermal solution in high-capacity CPO systems. Aiming at the heat dissipation requirements of the CPO system, this paper designs a liquid-cooled heat dissipation structure. And the incompressible steady Navier–Stokes equation is used to solve the flow and heat transfer in microchannels to optimize the heat dissipation structure. The simulation results show that, at a flow rate of 4 L/min, the maximum junction temperature of the 750 W switch chip is 97.3 °C. The maximum junction temperature of the surrounding optical modules is 31.3 °C, and the temperature difference is within 1.2 °C. And the experimental results show that the temperature simulation difference is within 4% and the pressure change trend is consistent with the simulation. Combining the experimental data and simulation results, the designed heat sink can satisfy the heat dissipation demands of the 51.2 Tbit/s bandwidth CPO system.

## Thermal requirements and theoretical methods

### Thermal requirements

**Fig. 1:**
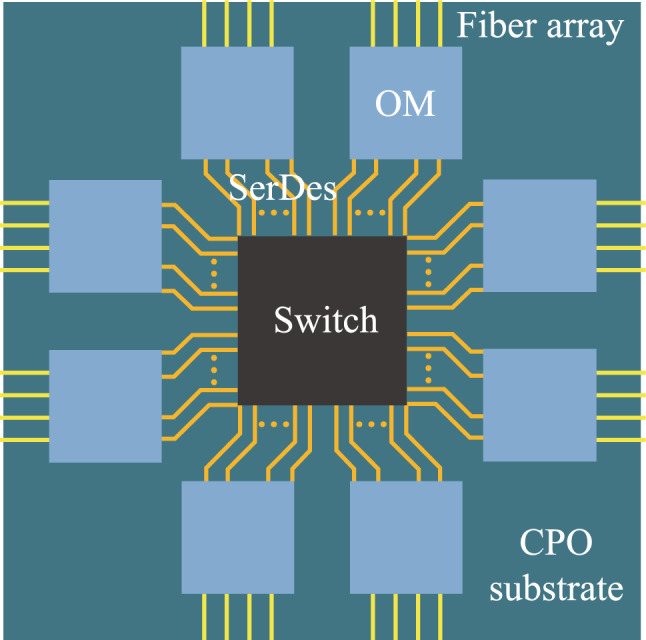
A CPO system with one switch chip and eight optical modules

As shown in Fig. 1, the CPO model discussed in this study consists of a switch chip and eight optical modules, which are integrated on the same substrate. The high-speed communication between the switch chip and optical modules via SerDes (Serializer/Deserializer) and the optical modules are connected to the external via fiber arrays.

As the bandwidth of the switch continues to increase, the power of the switch chip and optical module also increases. In a CPO system with a bandwidth of 51.2 Tbit/s, the power consumption of its switch chip can reach 750 W, and the heat flux is as high as 1.79 W/mm^2^. The heat flux of the optical module in the same system is about 0.2 W/mm^2^, which is much lower than that of the switch chip. Therefore, the switch chip requires a heat sink with stronger heat dissipation capability to ensure its normal operation. Referring to the maximum temperature target set by Intel, the junction temperature of the switch chip needs to be lower than 105 °C and the junction temperature of the optical module needs to be lower than 100 °C [4].

Given the proximity of the optical module to the switch chip and the temperature sensitivity of the optical devices within the module, the high heat flux generated by the switch chip may affect the functionality of the optical module. Consequently, in the design of thermal management, it is important to block the heat conduction path of the switch chip to avoid its interference with optical modules.

In addition, temperature uniformity between optical modules is a critical point in the design. The operating wavelength of an optical modulator will be shifted with the change of operating temperature. Currently, the mainstream optical modulators can be categorized into Mach − Zehnder modulators (MZMs) [28] and micro-ring modulators (MRMs) [29]. Among them, MRMs are more sensitive to temperature variations due to their precise structure. It has been shown that for every 1 °C increase in operating temperature, the operating wavelength of the MRM is shifted by approximately 0.1 nm [30]. In dense wavelength division multiplexing (DWDM) systems, the wavelength spacing is usually between 0.4 nm and 0.8 nm. Excessive temperature difference increases the insertion loss and may lead to crosstalk between optical channels. To ensure the stable operation of the data switching, the temperature difference of optical modules should be strictly controlled within 4 °C.

Based on the above analysis, the requirements of the CPO system can be summarized as follows:

High-efficiency cooling requirements for the switch chip: As the system bandwidth increases, the power density of the switch chip continues to increase, resulting in a significant increase in heat flux. Therefore, the heat sink must have sufficient heat dissipation capability to ensure that the temperature of the switch chip remains within a reasonable range. The junction temperature of the switch chip needs to be lower than 105 °C and the junction temperature of the optical module needs to be lower than 100 °C.

Avoiding the thermal crosstalk between the switch chip and optical modules: Since the optical module and the switch chip are integrated on the same substrate, the distance between them is relatively close. The heat generated by the switch chip may have a negative impact on the operation of the optical module.

Temperature uniformity requirements between optical modules: It is necessary to dissipate heat uniformly for all optical modules at the same time. To ensure the stable operation of optical chips, the maximum temperature difference between optical chips should be less than 4 °C.

### Theoretical methods

In fluid mechanics, Reynolds number can be used to determine the flow state of the fluid. In this study, the Reynolds number is defined by

1 $$\begin{array}{c}Re=\frac{\rho u{D}_{\text{H}}}{\mu },\end{array}$$

where *ρ* is the density of the fluid (kg/m^3^), *u* is the mean velocity of the fluid (m/s), *µ* is the dynamic viscosity of the fluid (kg/(m·s)), *D*_H_ is the hydraulic diameter of the pipe (m) and defined by

2 $$\begin{array}{c}{D}_{\text{H}}=\frac{2WH}{W+H},\end{array}$$

where *W* is the width of the pipe, *H* is the height of the pipe. According to the calculation of the Reynolds number, the fluid is laminar when the inlet flow rate is lower than 6 L/min.

This paper uses the Navier−Stokes equations to solve flow and heat transfer problems in microchannels [31, 32]. The flow is assumed to be (1) steady-state; (2) incompressible in three dimensions; (3) laminar; and (4) constant fluid properties. The governing equations are as follows.

Continuity equation

3 $$\begin{array}{c}\nabla \cdot \overrightarrow{u}=0.\end{array}$$

Momentum equation

4 $$\begin{array}{c}\rho \left(\overrightarrow{u}\cdot \nabla \right)\overrightarrow{u}=-\nabla p+\mu {\nabla }^{2}\overrightarrow{u}.\end{array}$$

Energy equation

5 $$\begin{array}{c}\rho C\left(\overrightarrow{u}\cdot \nabla T\right)=\lambda {\nabla }^{2}T,\end{array}$$

where *C* is the specific heat (J/(kg⋅K)), *λ* is the fluid thermal conductivity (W/(m⋅K)). The 6 sigmaET software is used to solve the model, and the convergence of the results was judged by monitoring the residues of continuity, temperature, and pressure drop.

## Simulation and optimization

**Fig. 2:**
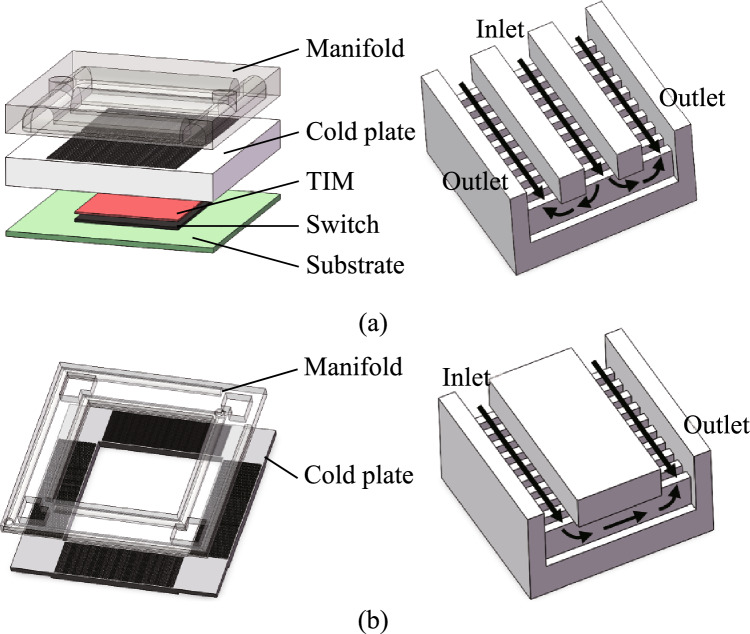
Cooling structures. **a** Heat sink of the switch chip. **b** Heat sink of optical modules

Based on the thermal requirements of the CPO system, this paper proposes a corresponding thermal management scheme. Considering the thermal crosstalk of the high-power switch chip to optical modules and the difference in the requirements of the switch chip and optical modules, two independent heat sinks are designed to meet the needs of two parts. The heat sink structures are shown in Fig. 2a and b.

For the optical module part, due to its lower heat flux, the design focuses on the temperature uniformity. Therefore, a parallel microchannel heat sink is used to ensure that the coolant flows into the microchannel cold plate of each optical module at the same temperature.

As for the switch chip, due to its extremely high heat flux, the TIM1.5 package is adopted [33]. And the manifold microchannel heat sink is used to cool the switch chip to ensure that its operating temperature is kept within a reasonable range. The microchannel cold plate has excellent heat dissipation performance, however, it can lead to a huge increase in the system pressure drop, which brings about additional pump power consumption. The use of manifolds can effectively reduce the system pressure drop, improve the overall system energy efficiency [34]. For the optical module part, due to its lower heat flux, the design focuses on the temperature uniformity. Therefore, a parallel microchannel heat sink is used to ensure that the coolant flows into the microchannel cold plate of each optical module at the same temperature. Considering that the cold plate of optical modules has shorter microchannels and more microchannels leading to a small flow rate of a single microchannel, so instead of using a 1-in-2-out manifold, simple straight channels are used to control the inflow and outflow of coolant.

**Fig. 3:**
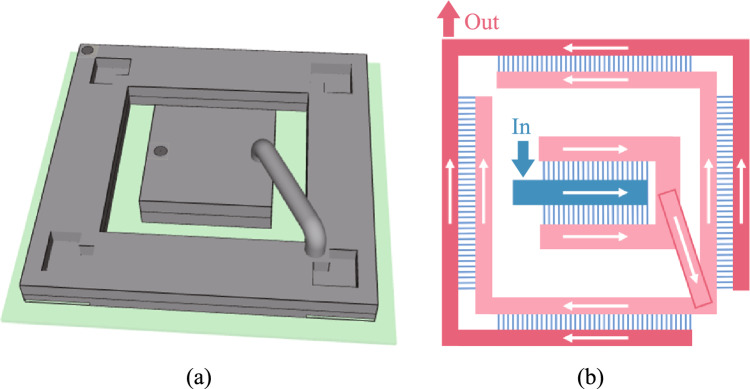
**a** Diagram of the assembly. **b** Schematic diagram of flow path layout

Figure 3a shows the overall structure of the heat dissipation system. The layout of the flow paths of the system is shown in Fig. 3b. The coolant first flows in from the manifold inlet of the switch chip heat sink. After sufficiently cooling the switch chip, the coolant will flow into the heat sink of optical modules. In the optical module heat sink, the coolant flows into the cold plate microchannel of each optical module at the same inlet temperature. It ensures the temperature uniformity of optical modules. Finally, the coolant flows out through the outlet of the optical module heat sink to complete the heat dissipation cycle.

### Thermal resistance model

**Fig. 4:**
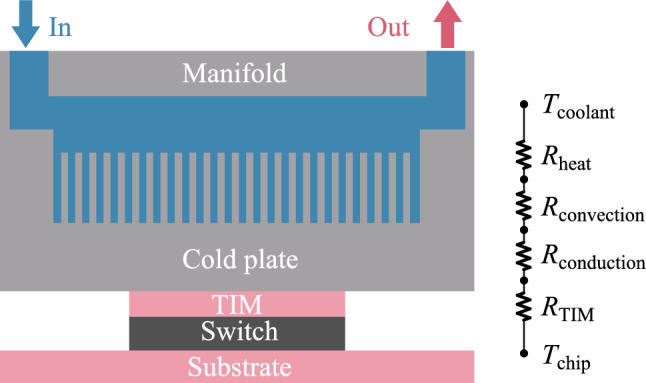
Structure of the TIM1.5 package and its one-dimensional thermal resistance model

Figure 4 illustrates the TIM1.5 structure of the switch chip. Many AI modules use the TIM1.5 packaging method, in which the chip and the heat sink are attached directly using TIM (Thermal Interface Material) [35]. This makes the heat transfer more efficient and gets rid of the need for reflow. However, this structure requires highly precise control during fabrication and has high reliability requirements [36]. A similar one-dimensional thermal resistance model of this structure is shown in Fig. 4. It shows the path of heat flux between the chip and the coolant [37].

Here, *T*_chip_ is the junction temperature of the chip, and *R*_TIM_ is the thermal resistance of TIM. *T*_coolant_ is the inlet temperature of the coolant, which is 20 °C in this paper.

*R*_conduction_ is the thermal resistance due to heat passing through the bottom of the cold plate, and is defined as:

6 $$\begin{array}{c}{R}_{\text{conduction}}=\frac{\tau }{\lambda A}+{R}_{\text{sp}},\end{array}$$

where *τ* is the thickness of the cold plate, *λ* is the thermal conductivity of the cold plate material, and *A* is the contact area of the chip. *R*_sp_ is the thermal spreading resistance. In electronic devices, heat usually diffuses from a small heat source region to a larger area, which can lead to significant thermal spreading resistance [38].

*R*_convection_ is the thermal resistance due to convective heat transfer between the solid material and the fluid, and is defined as:

7 $$\begin{array}{c}{R}_{\text{convection}}=\frac{1}{h{A}_{\text{convection}}},\end{array}$$

where *h* is the convective heat transfer coefficient, *A*_convection_ is the effective heat exchange area.

*R*_heat_ is the thermal resistance due to the water heating up as it absorbs heat, and is defined as:

8 $$\begin{array}{c}{R}_{\text{heat}}=\frac{1}{\rho Cf},\end{array}$$

where *f* is the flow rate. When *P*, the thermal design power consumption of the chip, is determined, the temperature difference between the coolant and the chip junction temperature can be calculated by combining all series thermal resistances:

9 $$\begin{array}{c}\Delta T=P\left({R}_{\text{TIM}}+{R}_{\text{conduction}}+{R}_{\text{convection}}+{R}_{\text{heat}}\right).\end{array}$$

### Boundary conditions

**Fig. 5:**
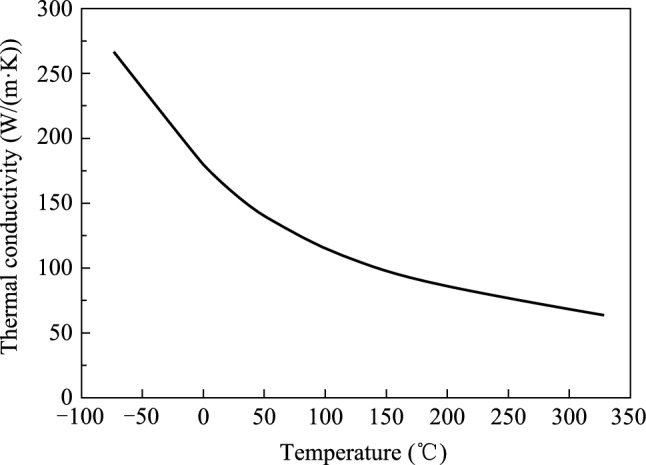
Variation of thermal conductivity of silicon with temperature

A simulation model is established according to the designed thermal management scheme. The coolant is deionized water, and the inlet temperature is 20 °C. The switch chip size is 25 mm × 16 mm, and the optical module size is 16 mm × 16 mm. The heat sink material is copper, and the substrate material is FR4. The chip is equated to a silicon block, and the ball grid array (BGA) is equated to an alloy block, and the material is typical solder. The thermal interface material (TIM) used to connect the heat sink to the chip is thermal grease with a bond line thickness of about 120 µm. The thermal conductivity [39] of all the materials used is shown in Table 1 (see Fig. 5).

**Table 1:** Thermal conductivity of materials

Material	Thermal conductivity (W/(m⋅K))
Water	0.612
Copper	386
Silicon	As shown in Fig. 5
Typical solder	46
Thermal grease	5.2

The CPO in the simulation model consists of one switch chip as well as eight optical modules, and the corresponding switch chip and optical module power consumption for each bandwidth is shown in Table 2. The parameters of each chip refer to the power consumption development forecast of switch chips published by the Optical Internetworking Forum (OIF) in 2022 [40]. The power consumption of many switch chips in the actual market at the corresponding bandwidth has exceeded this prediction. In this paper, the power consumption parameters are adjusted according to the actual situation to accurately reflect the actual demand [41]. The heat flux of the 51.2 Tbit/s switch chip is extremely high at 1.79 W/mm^2^, so the thermal structure that meets the thermal requirements of this CPO model can be applied to any other CPO systems.

**Table 2:** Chip power consumption at each bandwidth

Bandwidth (Tbit/s)	12.8	25.6	51.2
P-switch (W)	300	550	750
P-OM (W)	36	45	64

**Fig. 6:**
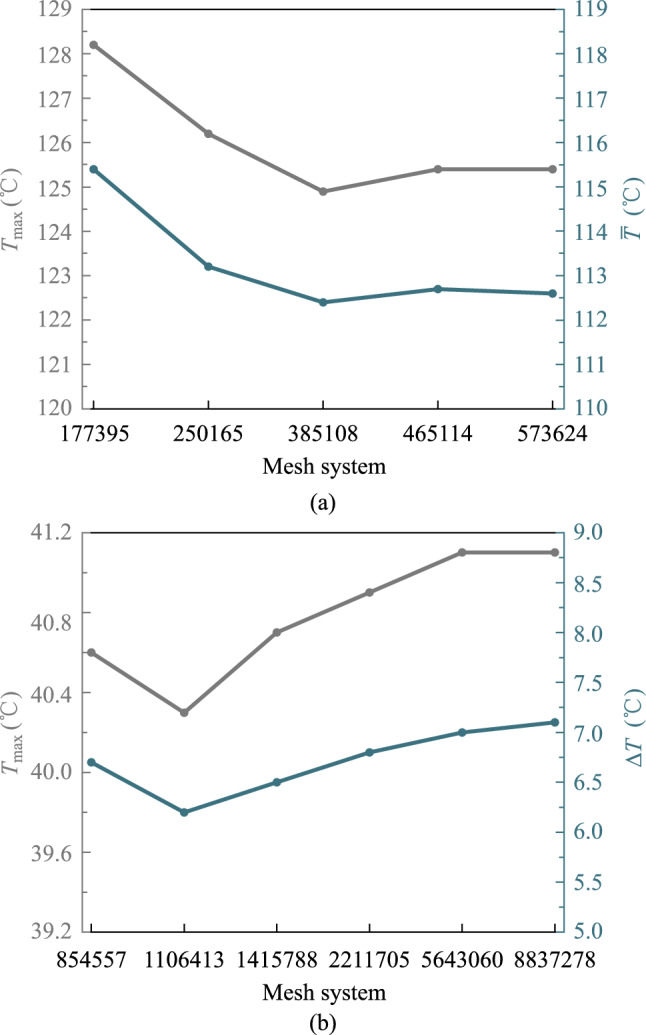
Variation of simulation temperatures with mesh system of **a** the switch chip heat sink, and **b** the optical module heat sink

Before performing the simulation optimization, based on the above model, the mesh was optimized. As shown in Fig. 6, the solution results have converged when there are 573,624 meshes for the switch chip heat sink and 8,837,278 meshes for the optical module heat sink.

### Optimization

Analyzing the established thermal resistance model, the junction temperature of the chip is closely related to the thickness of the cold plate, the effective heat exchange area of the microchannel cold plate, and the inlet flow rate. Among them, the effective heat exchange area is mainly related to the depth and width of microchannels. Therefore, heat sinks will be optimized based on these factors.

In the optimization, the junction temperature of the chip is seen as the main index. And with the increased switch bandwidth, the energy consumption problem in data centers becomes outstanding. A high system pressure drop leads to an increase in power consumption of the pump, which causes additional power loss. Therefore, the pressure drop is also considered one of the reference indicators in the optimization process.

The first step is to determine the inlet flow rate of the system. The relationship between chip junction temperature and pressure drop with respect to inlet flow rate was examined in the 25.6 Tbit/s CPO.

**Fig. 7:**
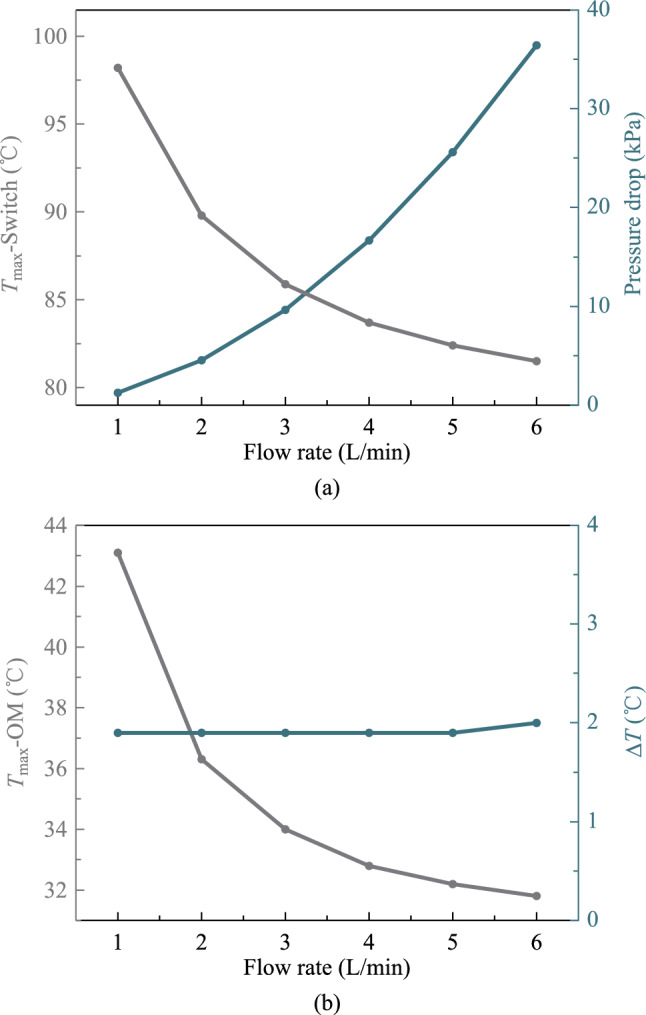
**a** Trend of maximum switch chip temperature and the system pressure drop with flow rate. **b** Trend of maximum temperature and temperature difference of optical modules with flow rate

Figure 7 illustrates that the decrease rate of the switch chip junction temperature gradually slows down as the flow rate increases, yet the pressure drop of the system increases exponentially. The maximum junction temperature among optical modules also decreases with the increase in flow rate, and its decrease also slows down gradually. The maximum temperature difference between the optical modules remains basically constant. Considering these factors, 4 L/min is the optimal inlet flow rate.

After determining the inlet flow rate, parameters of the heat dissipation structure will be optimized based on its performance in a 51.2 Tbit/s CPO system. Owing to the significant difference in cooling demands between the switch chip and the optical module, the two heat sinks will be optimized independently instead of optimizing them as a unified system.

#### Optimization of switch heat sink

**Fig. 8:**
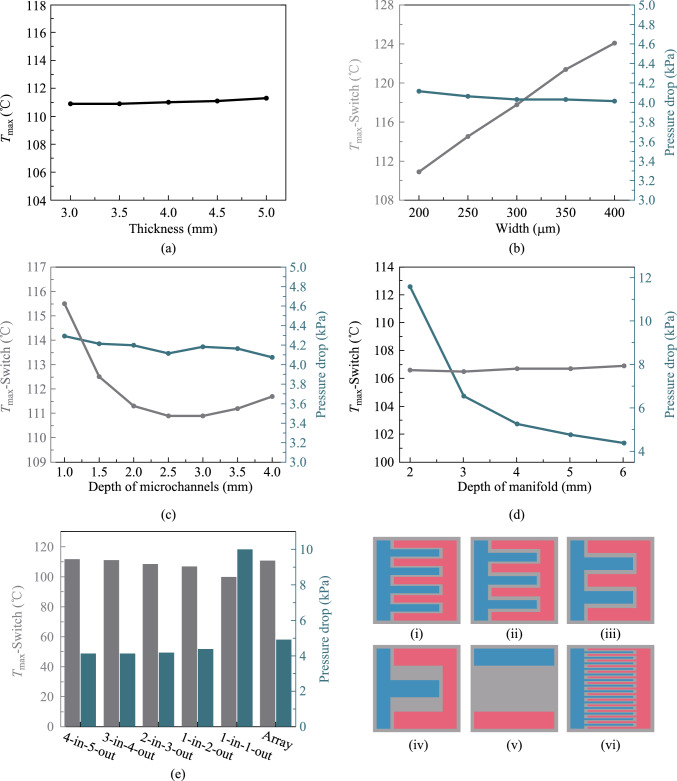
Optimization of structural parameters of switch chip heat sink. **a** Variation of maximum chip junction temperature with cold plate thickness. **b** Variation of maximum chip junction temperature and pressure drop with microchannel width. **c** Variation of maximum chip junction temperature and pressure drop with microchannel depth. **d** Variation of maximum chip junction temperature and pressure drop with manifold channel depth. **e** Effect of different manifold layouts on junction temperature and the pressure drop: (i) 4-in-5-out, (ii) 3-in-4-out, (iii) 2-in-4-out, (iv) 1- in-2-out, (v) 1-in-1-out, and (vi) array

In this part, the switch chip power is set to 750 W. Figure 8 shows that the thickness of the cold plate does not have a significant effect on the chip junction temperature. However, the thickness of the contact layer should not be too thin considering the possible reliability hazards caused by thermal stress.

Numerous studies have investigated the effect of the ratio of fin width to channel width in high depth-to-width ratio microchannels and found that a ratio of 1:1 usually results in an optimal thermal distribution [42–44]. Consequently, the microchannel width is consistently equivalent to the fin width in this study. From Fig. 8b, the junction temperature dramatically reduces with a reduction in microchannel width, which indicates that the width of the microchannel considerably influences the heat dissipation efficiency of the heat sink. The pressure drop increases slightly as the microchannel width decreases, which is a justifiable trade-off relative to the enhancement in heat dissipation efficiency.

Nonetheless, due to manufacturing challenges and reliability concerns, the fin width cannot be lowered indefinitely. The definitive microchannel width is 200 µm.

The microchannel depth significantly influences the chip junction temperature. Insufficient depth results in a limited effective heat exchange area between the cold plate and the coolant. When the depth is excessive, the heat transfer efficiency at the channel’s bottom may decrease, while the heat transfer contribution from the top of the fins will also decrease, which also leads to a decline in the heat dissipation efficiency. The pressure drop in the heat sink remains relatively constant, despite variations in the microchannel depth.

The effect of manifold depth on the pressure drop is more pronounced. With the increased manifold depth, the pressure drop decreases rapidly. And the junction temperature increases slightly with the increased manifold depth.

In addition to optimizing the heat sink parameters, different structures are concerned with improving the thermal performance. The manifold design substantially influences the heat dissipation effect. As can be seen from Fig. 8e, the 1-in-1-out manifold layout has the best heat dissipation efficiency, but it incurs a markedly higher pressure drop compared to other designs. The 1-in-2-out manifold ranks just below the 1-in-1-out manifold regarding heat dissipation efficiency with a slight increase in pressure drop.

Furthermore, different microchannel structures are tried to further improve the heat transfer performance. To enhance the heat transfer performance of liquid cold plates, more complex microchannel structures have been applied to cold plate cooling. This study involves microchannels with wavy shapes, as well as those with dimpled structures or needle ribs [45, 46].

**Fig. 9:**
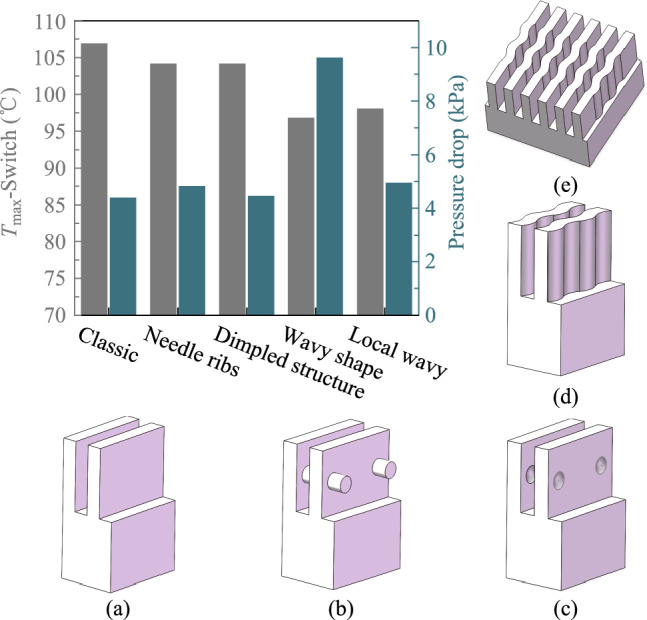
Thermal performance of switch chip heat sinks with different microchannel shapes. **a** Conventional straight microchannel. **b** Microchannel with needle ribs. **c** Microchannel with dimpled structures. **d** Microchannel with wavy shapes. **e** Microchannel with local wavy shapes

Comparing with the traditional long, straight microchannels, these microchannels with new shapes help to disturb the boundary layer at the bottom of the channel and enhance the heat transfer efficiency. By using 6 sigmaET software, the turbulence model is solved. The results are shown in Fig. 9.

In comparison to the conventional straight microchannel, the novel microchannel structure markedly enhances heat transfer efficiency. With the microchannel with needle ribs or with dimpled structures, there is no significant change in the pressure drop, while there is a small decrease in the chip junction temperature. The microchannel with wavy shapes exhibits the most effective heat dissipation. However, at a flow rate of 4 L/min, the pressure drop is twice that of the other structures. Therefore, we designed a local wavy microchannel, implementing the wavy shape only in the hotspot region at the center of the chip, while the rest still utilizes the straight channel. This structure not only offers superior heat dissipation while maintaining pressure drop increases within an acceptable range. The length of the wavy channel can be modified based on the heat dissipation requirements. Increased length enhances heat dissipation capacity, but the pressure drop increases accordingly.

#### Optimization of optical module heat sink

**Fig. 10:**
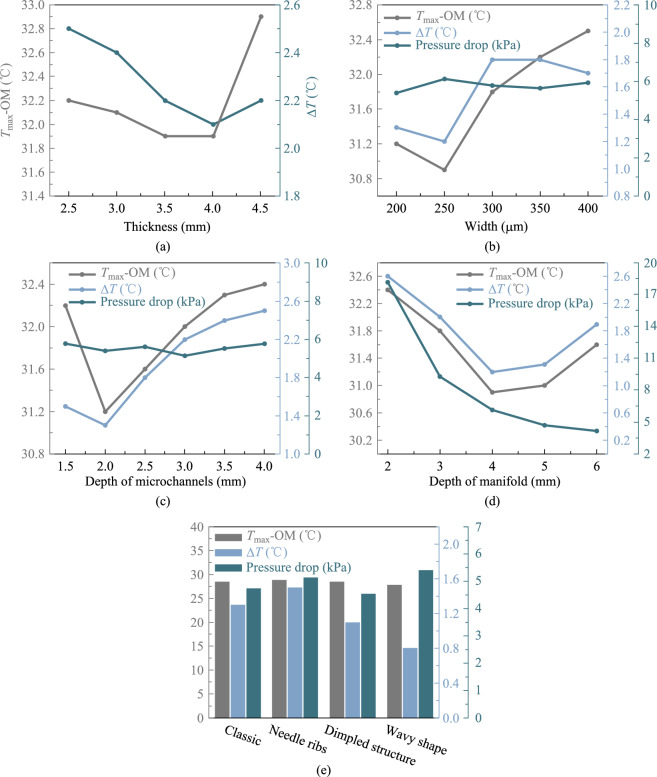
Optimization of structural parameters of optical module heat sink. **a** Variation of maximum junction temperature and temperature difference of optical modules with cold plate thickness. **b** Variation of maximum junction temperature, temperature difference, and pressure drop with microchannel width. **c** Variation of maximum junction temperature, temperature difference, and pressure drop with microchannel depth. **d** Variation of maximum junction temperature, temperature difference, and pressure drop with manifold channel depth. **e** Effect of microchannel with different shapes on maximum junction temperature, temperature difference, and the pressure drop

The CPO system includes 8 optical modules, each with a power consumption of 64 W. Figure 10 shows that the maximum junction temperature and the temperature uniformity of the optical modules are affected by the thickness of the cold plate in the optical module heat sink. The optimal heat dissipation occurs with a cold plate thickness of 4 mm.

The microchannel width shows a certain relationship with the junction temperature. As the width decreases, the junction temperature progressively declines. However, when the microchannel width is reduced to 200 µm, the junction temperature increases, contrasting with the behavior observed in the switch chip heat sink. The cause may be the low power consumption of the optical module. The contact area of the cold plate with a 250 µm microchannel width is enough to satisfy the heat dissipation demand. In addition, the reduction of the microchannel width affects the distribution of fluid in the channel, resulting in uneven heat dissipation. Similar to the switch chip heat sink, variations in microchannel width exert minimal influence on pressure drop.

The microchannel depth of the optical module heat sink follows a similar trend to that of the switch chip heat sink. The best heat dissipation occurs when the microchannel depth is around 2 mm. The change in microchannel depth still has little effect on the pressure drop. The change in manifold depth has a greater effect on the pressure drop, which gradually decreases as the manifold depth increases. A shallow manifold depth results in narrow flow channels, hence increasing the pressure drop. Conversely, an excessively deep manifold reduces the pressure drop and correspondingly diminishes heat dissipation capacity.

The wavy microchannel in the optical module heat sink exhibits superior heat dissipation compared to other designs, while the increase in pressure drop remains within an acceptable range. This is due to the shorter microchannel and the large number of microchannels in the optical module heat sink, which results in a smaller flow rate in each channel.

### Results

After completing the optimization of the two heat sinks, the thermal performance of optimized heat sinks in the assembled system. Table 3 presents the comparison of simulation results before and after optimization.

**Table 3:** The comparison of simulation results before and after optimization

	*T*_max_-Switch (°C)	*T*_max_-OM (°C)	∆*T*-OM (°C)	Pressure drop (kPa)
Before	121	33	2.4	16.703
After	97.3	31.3	1.2	17.056

After optimization, the junction temperature of the switch chip is significantly decreased to 97.3 °C, the maximum junction temperature of the optical modules is decreased to 31.3 °C, and the temperature difference between the optical modules is reduced from the initial 2.4 °C to 1.2 °C. While the performance is greatly optimized, the pressure drop remains nearly constant. The results indicate that the optimized thermal structure can well satisfy the thermal demands of the 51.2 Tbit/s CPO system.

## Experiment

### Experimental platform

This study imitates the CPO system by flip-bonding the thermal test chip to the test board. The heat sinks are fabricated using direct metal laser sintering (DMLS) technology, through which the heat sink is fabricated in an integrated manner, which can ensure the sealing of the heat sink and effectively avoid the problem of liquid leakage.

**Fig. 11:**
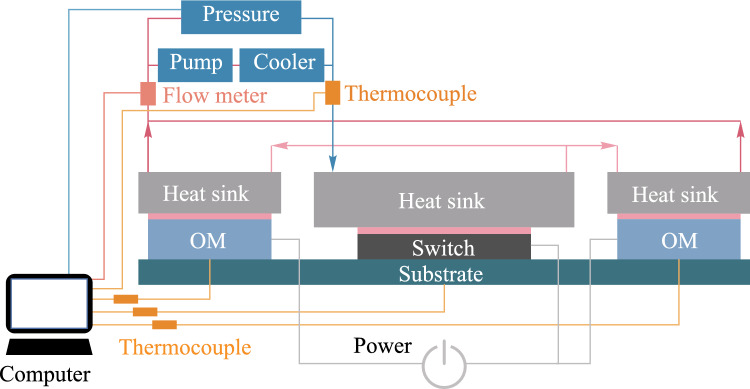
Schematic diagram of experimental platform

**Fig. 12:**
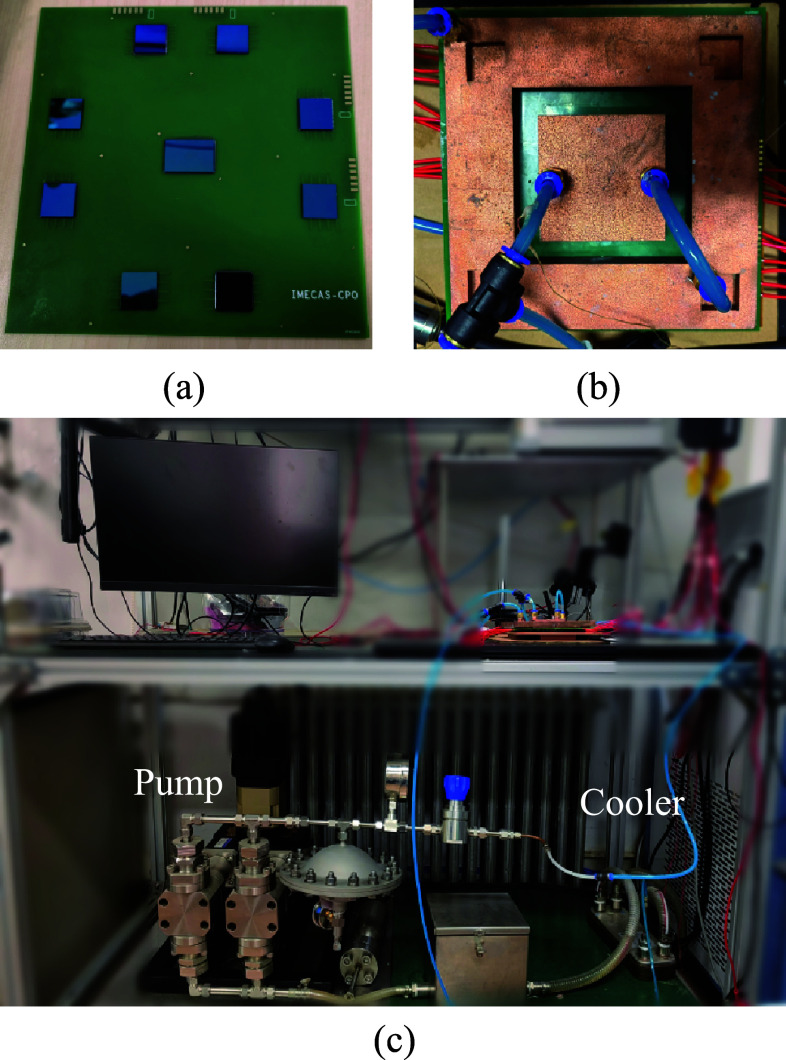
**a** Test board. **b** Heat sinks installation. **c** Constructed experimental platform

Figure 11 is the schematic diagram of the experimental platform. First, the heat sinks were connected to the CPO chips via TIM. Subsequently, a pump and a cooler are connected to form a circulating heat dissipation system. A differential pressure gauge is connected to the inlet and outlet ports to measure the pressure drop in the system. The thermocouple probes were inserted into the bottom center of the chips to monitor the junction temperature of the chips. There also exist thermocouples at the inlet and outlet ports to monitor the change of fluid temperature. A flow meter was installed at the water inlet hose to monitor the pressure drop change. During the experiment, the computer system can display and record the changes in temperature, flow rate, and pressure drop data in real time. The constructed experimental platform is shown in Fig. 12.

### Experimental results

**Fig. 13:**
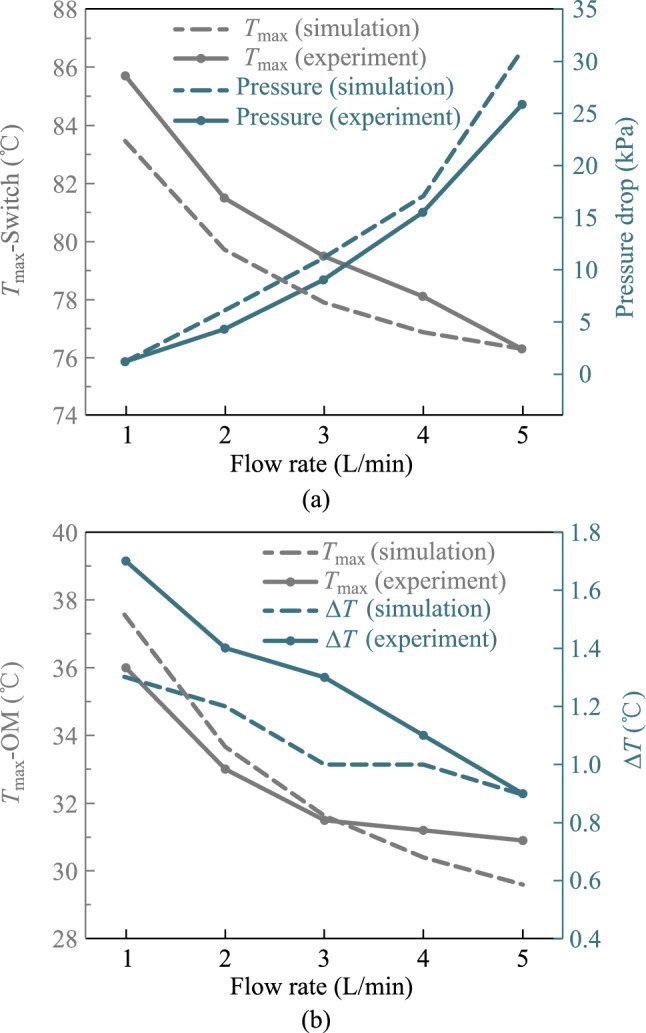
**a** Variation of maximum switch chip junction temperature and the system pressure drop with flow rate. **b** Variation of maximum junction temperature and temperature difference of optical modules with flow rate

From Fig. 13a, the trends of the switch chip junction temperature and the system pressure drop with flow rate in the 25.6 Tbit/s CPO system are consistent with the simulation. When the flow rate is 1 L/min, the maximum deviation between the simulated and experimental results is 2.3 °C. The difference is within 4%, which can prove the reliability of the simulation results. The overall pressure drop of the system is lower than the simulation value, but it still increases exponentially with the flow rate. And analyzing the results, 4 L/min is a suitable flow rate. The results in Fig. 13b show that the maximum deviation of the maximum junction temperature of optical modules is within 1.5 °C, which proves that the model has high reliability in temperature simulation. The temperature difference of the optical modules is slightly higher than the simulation value, possibly because the heat sink installation cannot guarantee the same thermal grease thickness. However, even at a flow rate of 1 L/min, the maximum temperature difference does not exceed 1.7 °C, which proves that the structure has excellent temperature uniformity performance.

**Fig. 14:**
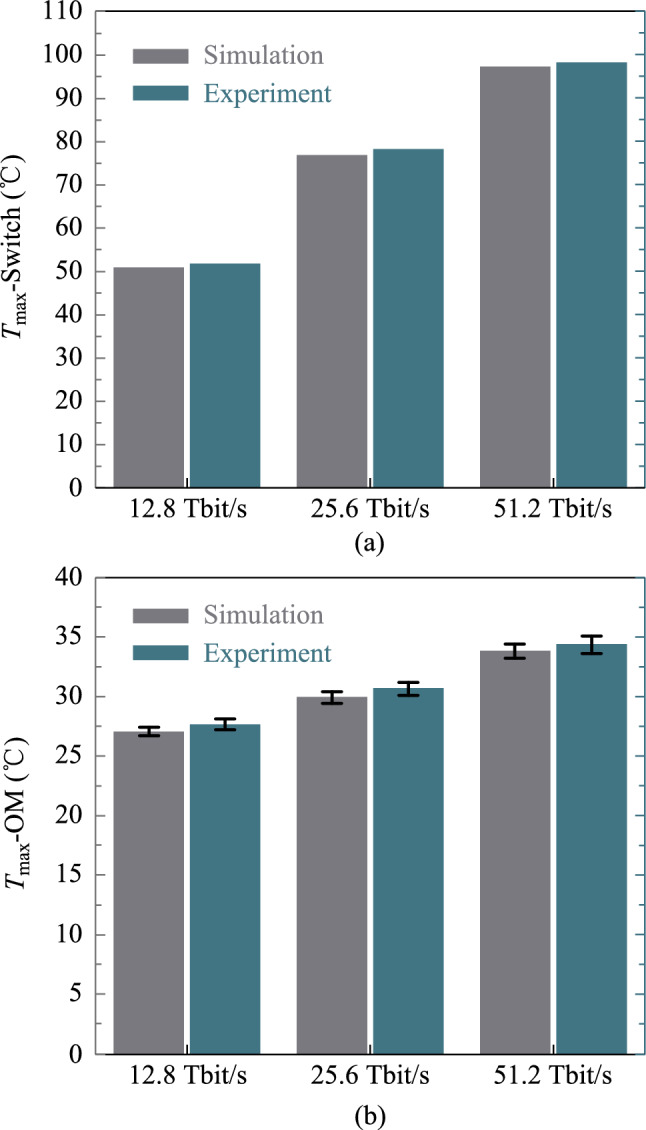
**a** Maximum switch chip junction temperature and the system pressure drop in the CPO system with different bandwidths. **b** Maximum temperature and temperature difference of optical modules in the CPO system with different bandwidths

Figure 14 depicts the operation of this heat dissipation structure in the CPO systems of 12.8 Tbit/s, 25.6 Tbit/s, and 51.2 Tbit/s with the inlet flow rate of 4 L/min. The experimental results are basically consistent with the simulation results. When the CPO bandwidth reaches 51.2 Tbit/s, the maximum temperature of the switch chip is 98.3 °C, and the maximum temperature of the optical modules is 35.1 °C, with a maximum temperature difference of 1.5 °C. The structure performs well in cooling the high heat flux switch chip and maintaining the homogeneous temperature of the optical modules and can be well applied to the thermal management of the 51.2 Tbit/s CPO system.

## Conclusion

This study examines the implementation of liquid-cooled heat dissipation in CPO systems. Considering its special structure and application scenario, we analyzed the heat dissipation requirements and indexes of CPO. The high power consumption of the switch chip necessitates careful attention to thermal crosstalk blocking. In addition, uniform heat dissipation of the optical modules is required to ensure signal quality and reduce the bit error ratio (BER) of the system.

After requirement analysis, a heat dissipation structure applied to CPO is designed. A discrete heat dissipation structure is adopted to avoid the switch chip affecting the work of optical modules. We use a manifold microchannel heat sink to dissipate heat from the high-power consumption switch chip. Using a local wavy-shaped microchannel cold plate improves the thermal performance without any additional pump power consumption. In the optical module part, a parallel microchannel structure realizes temperature homogeneity. Simulation results show that the junction temperature of the switch chip is 97.3 °C when its power reaches 750 W, and the maximum temperature difference of the optical modules in the system is 1.2 °C. The experiment is conducted to verify the reliability of the simulation results and proves that the heat dissipation structure can be applied to the heat dissipation of the 51.2 Tbit/s CPO system.

It is believed that the application of liquid-cooled heat dissipation technology in CPO is promising and has the potential to become one of the key technologies in the thermal management of server clusters in future data centers.

## Data Availability

The data that support the findings of this study are available from the corresponding author, upon reasonable request.
